# Standardized protocol for quantification of nerve bundle density as a biomarker for endometriosis

**DOI:** 10.3389/frph.2023.1297986

**Published:** 2023-11-30

**Authors:** Gerbrand Zoet, Dwayne R. Tucker, Natasha L. Orr, Fahad T. Alotaibi, Yang Doris Liu, Heather Noga, Martin Köbel, Paul J. Yong

**Affiliations:** ^1^ Department of Obstetrics and Gynecology, University Medical Center Utrecht, Utrecht, Netherlands; ^2^ Department of Obstetrics and Gynecology, University of British Columbia, Vancouver, BC, Canada; ^3^ University of British Columbia Endometriosis and Pelvic Pain Laboratory, Vancouver, BC, Canada; ^4^ Department of Physiology, College of Medicine, Imam Mohammad Ibn Saud Islamic University (IMSIU), Riyadh, Saudi Arabia; ^5^ Women’s Health Research Institute, Vancouver, BC, Canada; ^6^ Department of Pathology and Laboratory Medicine, University of Calgary, Calgary, AB, Canada

**Keywords:** endometriosis, PGP9.5, nerve, immunohistochemistry, pain

## Abstract

**Introduction:**

We propose a standardized protocol for measurement of nerve bundle density in endometriosis as a potential biomarker, including in deep endometriosis (DE), ovarian endometriomas (OMA) and superficial peritoneal endometriosis (SUP).

**Methods:**

This was a prospective cohort of surgically excised endometriosis samples from Dec 1st 2013 and Dec 31st 2017 at a tertiary referral center for endometriosis in Vancouver, BC, Canada. Surgical data were available from linked patient registry. Protein gene product 9.5 (PGP9.5) was used to identify nerve bundles on immunohistochemistry. PGP9.5 nerve bundles were counted visually. To calculate nerve bundle density, PGP9.5 nerve bundle count was divided by the tissue surface area (total on the slide). All samples were assessed using NHS Elements software for semi-automated measurement of the tissue surface area. For a subset of samples, high power fields (HPFs) were also counted as manual measurement of the tissue surface area. Intraclass correlation was used to assess intra observer and inter observer reliability. Generalized linear mixed model (GLMM) with random intercepts only was conducted to assess differences in PGP9.5 nerve bundle density by endometriosis type (DE, OMA, SUP).

**Results:**

In total, 236 tissue samples out of 121 participants were available for analysis in the current study. Semi-automated surface area measurement could be performed in 94.5% of the samples and showed good correlation with manually counted HPFs (Spearman's rho = 0.781, *p* < 0.001). To assess intra observer reliability, 11 samples were assessed twice by the same observer; to assess inter observer reliability, 11 random samples were blindly assessed by two observers. Intra observer reliability and inter observer reliability for nerve bundle density were excellent: 0.979 and 0.985, respectively. PGP9.5 nerve bundle density varied among samples and no nerve bundles could be found in 24.6% of the samples. GLMM showed a significant difference in PGP9.5 nerve bundle density between the different endometriosis types (X^2^ = 87.6, *P* < 0.001 after adjusting for hormonal therapy, with higher density in DE and SUP in comparison to OMA).

**Conclusion:**

A standardized protocol is presented to measure PGP9.5 nerve bundle density in endometriosis, which may serve as a biomarker reflecting local neurogenesis in the endometriosis microenvironment.

## Introduction

Endometriosis is characterized by the presence of endometrial-like tissue outside the uterus and affects approximately 10% of women and an unmeasured number of gender diverse people mainly during their reproductive age (1, 2). Endometriosis causes chronic and cyclic pelvic pain and may lead to infertility (3). Endometriotic lesions can spread throughout the pelvic cavity and there are three anatomical types: superficial peritoneal endometriosis (SUP), ovarian endometriomas (OMA), and deep endometriosis (DE) often found as nodules in the cul-de-sac between uterus and rectum. Pelvic pain symptoms vary among patients and do not correlate well with the often-used revised American Society of Reproductive Medicine (rASRM) staging classification (4). The complex relationship between endometriosis and pain symptoms is not fully understood.

Endometriotic lesions contain nerve fibers which are stimulated in an inflammatory cascade, leading to increased nociceptive response (1). Local neuroproliferation (or neurogenesis) is suggested to play a role as well, whereby there is a quantitative increase in nerve fibers around histologically confirmed endometriosis (5). For example, patients with deep dyspareunia had a higher density of bundles of nerve fibers around histologically confirmed endometriosis, than patients with endometriosis but without deep dyspareunia, (6, 7) with nerve growth factor (NGF) expression by endometriosis correlating with higher nerve bundle density (8). However, nerve fibers are not found in all endometriotic lesions (9–11). In addition, nerve bundle density and NGF can differ spatially within a surgical endometriotic lesion (12).

The results of these studies assessing nerve bundle density in endometriosis can be difficult to compare, due to different methods for measurement of nerve bundle density. In the current study, we propose a standardized protocol for measurement of nerve bundle density in endometriosis and assess variability in nerve bundle density among patients with endometriosis by anatomic type.

## Methods

### Setting

The current study is part of an ongoing prospective cohort of patients undergoing endometriosis surgery (including excision of lesions), conducted in the academic tertiary referral Centre for Pelvic Pain and Endometriosis within BC Women's Hospital in Vancouver, Canada. Ethical approval for biobanking was obtained from the University of British Columbia (H14-03040) and informed consent was obtained from patients. We examined nerve bundle density in available tissue blocks from patients in the cohort who underwent endometriosis surgery between Dec 1st 2013 and Dec 31st 2017. Exclusion criteria were malignancy or post-menopausal status (spontaneous or surgical). All anatomic types were included: DE, OMA and SUP. It should be noted that a single patient could have one to three of the anatomic types, and more than one lesion within each type. Linked clinical data from the patient were available from a prospective registry as described previously (H16-00264; Clinicaltrials.gov NCT02911090), (13) which included standardized surgeon-reported data at the time of surgery. This cohort has been described previously (14). For each tissue block from each patient included in the study, a tissue slide was cut for H&E and histological endometriosis was confirmed.

### Immunohistochemistry

**Figure 1:**
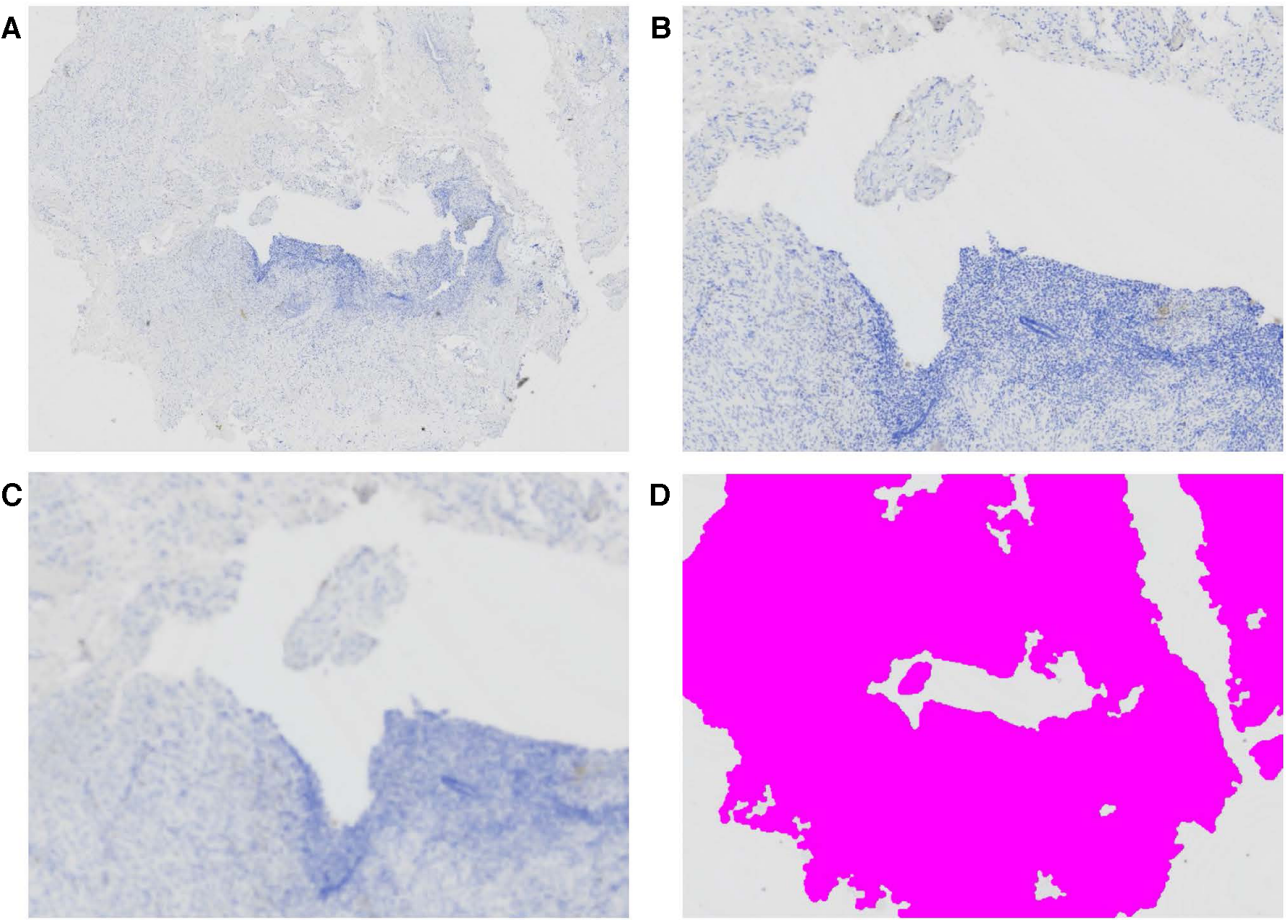
Semi-automated surface area measurement of endometriosis tissue sample. (**A**) Large image at 80× enlargement, (**B**) detail of central part of this tissue at 200× enlargement, (**C**) applying GA3 workflow to smoothen the image and (**D**) application of color based on intensity of the smoothened image, after removal artefacts.

Immunohistochemistry was done on cases grouped together after the biobanking was completed. From each tissue block with confirmed endometriosis, another tissue slide was cut and examined with a Nikon Eclipse Ni-E microscope (Nikon). The total tissue surface area of each slide was measured in a semi-automated fashion using NIS Elements software. An image was made using a 20× objective and a 4× ocular, creating an 80× enlargement. The image of the total surface of the lesion was saved and used for further analysis. Creating specific General Analysis (GA3) workflows enabled automated surface area measurement (see Figure 1, displaying the steps in surface area measurement). The first step in creating this GA3 workflow was to apply smoothening of the saved image. Secondly, all visible tissue was digitally colored based on the intensity of the measured signal. Afterward any artefacts which were falsely measured as tissue based on intensity were removed. Lastly, the surface of the colored area was computed by the software and displayed in µm^2^. Separate workflows were created for different endometriosis types, since signal density varied among DE, SUP, and OMA lesions. The appropriate workflow was chosen based on performance in measuring surface area.

Manual counting of the number of high-power fields (HPFs) was done for a subset of the OMA and SUP lesions, and correlated to the surface area from the semi-automated workflow.

The samples also underwent immunohistochemistry (IHC) with a mouse monoclonal anti-human PGP9.5 antibody (clone 10A1, Leica Biosystems, NCL-L-PGP9.5) as a pan-neuronal marker for nerve bundles around histological endometriosis on the tissue slide). IHC was performed on 4 µm sections with 30 min of pre-treatment heat-induced antigen retrieval in Tris-EDTA buffer, pH = 9.0; primary antibody incubation for 30 min at dilution 1/200, 10 min of a mouse linker, and 30 min for the peroxidase labelled Dako EnVision + polymer-based detection system (Dako protocol 1hr-10M-30, Agilent, Santa Clara, CA, USA).

**Figure 2:**
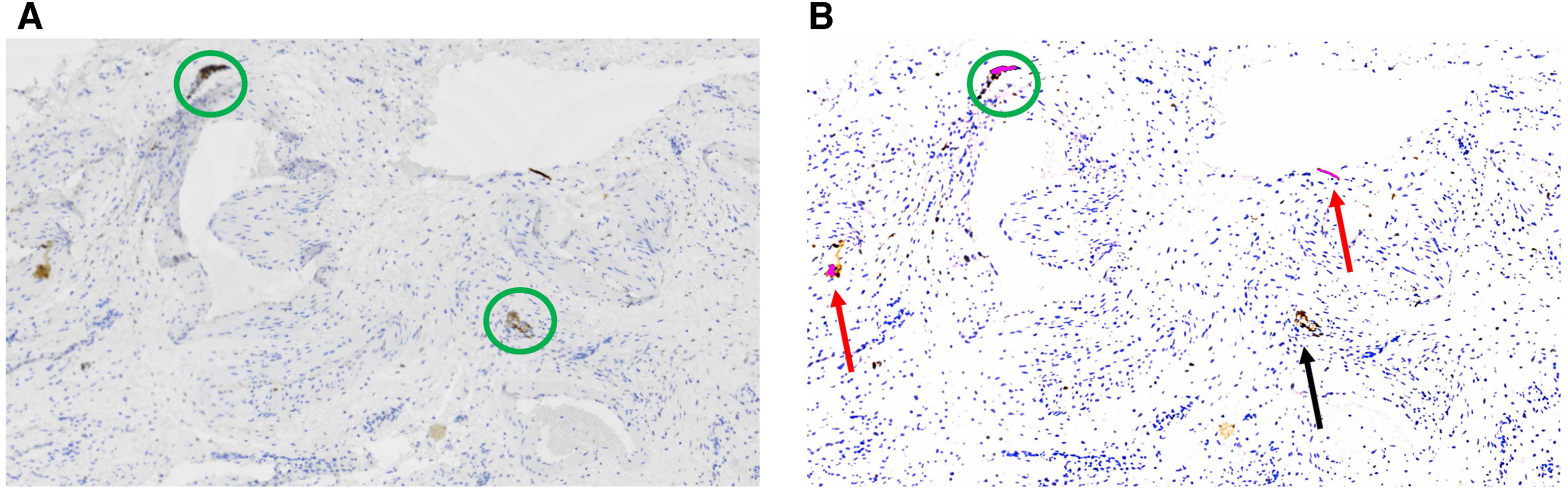
(**A**) In the original large image at 80× enlargement, nerve bundles are indicated by green circles, (**B**) in the smoothened image after applying the GA3 workflow, correctly counted nerve bundles are indicated in green circles, falsely counted artefacts are indicated by red arrows and falsely not counted nerve bundle is indicated by a black arrow.

We assessed PGP9.5 nerve bundles (i.e., bundles of PGP9.5 positive nerve fibers), similar to as described previously, (7) with or without surrounding perineurium. We attempted to create a separate GA3 workflow for PGP9.5 nerve bundle counts. However, due to the heterogeneity in the aspect of nerve bundles and the background scatter that often appeared to have the same size and signal density as nerve bundles, it was not possible to create a workflow that correctly counted all nerve bundles (Figure 2). Therefore, slides were manually scanned top to bottom and left to right, to count the total number of nerve bundles with a 20× objective and 10× ocular (200× HPF).

### Statistical analyses

The observers scoring the slides were blinded for other variables (anatomic type and all clinical data).

Spearman’s rank correlation was performed to assess the association between the semi-automated surface area measurement and manual counting of HPFs.

PGP9.5 positive nerve bundle density was computed by dividing the total nerve bundle count by the semi-automated surface area (µm^2^) and then multiplying it by 10^8^ for ease of interpretation. To assess intra observer and inter observer reliability, 11 samples were assessed each twice by one observer and 11 samples were assessed by two observers, respectively. Intraclass correlation estimates and their 95% confident intervals were used to assess intra observer reliability and inter observer reliability, and values >0.75 was considered reliable.

PGP9.5 nerve bundle density was a continuous variable with a proportion of slides having zero nerve density; moreover, each patient could have one or more endometriosis anatomic types and one or more sampled lesions per type. Therefore, we built a generalized linear mixed model (GLMM) with random intercepts only to assess differences in nerve bundle density scores. In the regression, PGP9.5 nerve bundle density was the response variable, endometriosis type (DE, OMA, and SUP) was the explanatory variable, and hormonal treatment before surgery (yes or no) was the covariate being adjusted for given its possible impact on nerve density around endometriosis (10, 15). A zero-inflated model was used to account for the fact that a sample could have zero nerve density. A mixed model approach allowed for sampling more than one lesion per patient. SUP lesions were used as reference group within the pairwise comparisons, allowing for comparison of DE to SUP and of OMA to SUP, but this methodology did not allow direct comparison between DE and OMA.

All statistical analyses were performed using SPSS 29.0 (IBM Corporation, Armonk, NY, USA) and SAS 9.4 (SAS Institute, Cary, NC, USA); *p* < 0.05 (two-tailed) was considered significant.

## Results

### Baseline characteristics

In total, 236 endometriosis tissue samples from 121 patients were included in the study. Sample characteristics are described in Table 1. Among the 121 patients, the mean age was 34.6 years and most participants were Caucasian (75.2%, 91/121) and nulliparous (72.5%, 87/120). Half of the participants used hormonal treatment right before they had their surgery (50.4%; 60/119, with missing hormonal data for 2 cases). Almost all patients had a laparoscopic approach (98.3%). rASRM stages were 24.6% (29/118) Stage I, 17.8% (21/118) Stage II, 22.0% (26/118) Stage III, and 35.6% (42/118) Stage IV. A variable number of tissue samples stained for PG9.5 nerve bundles was available, ranging from one to eight samples (blocks) per patient (Table 1). Of the 236 tissue samples, there were 120 DE, 46 OMA, and 70 SUP (although these are not mutually exclusive, because a single patient could have multiple lesions).

**Table 1:** Baseline characteristics (*n* = 121).

Variable	Percentage (count) or mean (SD)
Age (years)	34.5 (6.6)^a^
BMI (kg/m^2^)	25.3 (5.1)^b^
Caucasian ethnicity	75.2% (91/121)
Current smoker	13.3% (16/120)
Previous pregnancy	41.7% (50/120)
Previous delivery	27.5% (33/120)
Using hormonal treatment before surgery	50.4% (60/119)
Surgical procedure: laparoscopy	98.3% (119/121)
rASRM stage	
Stage I	24.6% (29/118)
Stage II	17.8% (21/118)
Stage III	22.0% (26/118)
Stage IV	35.6% (42/118)
Number of tissue samples per patient	
1 sample	52.1% (63/121)
2 samples	20.7% (25/121)
3 samples	11.6% (14/121)
4 samples	7.4% (9/121)
5 samples	5.8% (7/121)
6 samples	1.7% (2/121)
7 samples	0%
8 samples	0.8% (1/121)

SD, standard deviation; BMI, body mass index; rASRM, revised American society for reproductive medicine.

^a^ *n* = 120.

^b^ *n* = 119.

### Semi-automated surface area measurement

**Figure 3:**
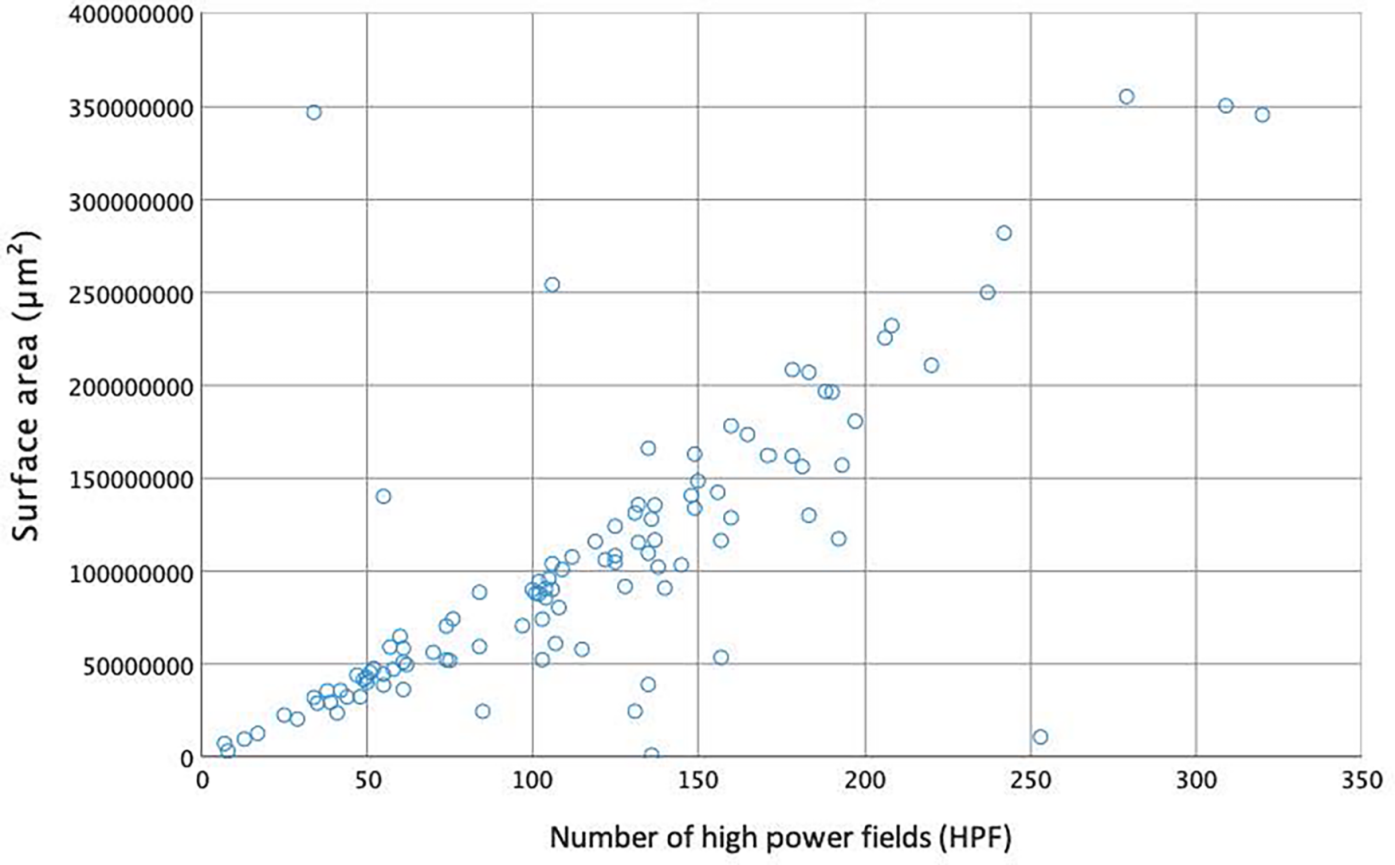
Correlation between semi-automated surface area measurement (μm^2^) and manual counted high power fields.

Semi-automated surface area measurement using GA3 workflow in NHS Elements software could be performed in 223 of the 236 samples (94.5%). Semi-automated surface area measurement was not feasible in the remaining 13 samples (5.5%). The automated surface area measurement was plotted against the manual HPF count, which showed a significant correlation: Spearman's rho = 0.781, *p* < 0.001, see Figure 3.

### PGP9.5 nerve bundle density

The PGP9.5 nerve bundle density was computed by dividing the number of PGP9.5 nerve bundles counted manually, by the automated surface area. The intra observer reliability (same rater assessing each sample, repeated twice per sample) and inter observer reliability (two raters assessing each sample) were excellent, with intraclass correlations of 0.979 (0.927–0.994) and 0.985 (0.943–0.996) respectively.

Some endometriosis tissue samples did not contain any nerve bundles. In 58 samples (24.6%) no nerve bundles could be found, which appeared to differ depending on the specific type of endometriosis lesion: the majority of the OMA (67.4%) showed no nerve bundles (31 out of 46 samples) whereas only a minority of the DE (18.3%) and SUP lesions (7.1%) did not contain any nerve bundles (22 out of 120 and 5 out of 70, respectively (see Table 2).

**Table 2:** Nerve bundle density score according to tissue type.

PGP9.5 nerve bundle density score (categorized)	Anatomic type (*n* = 236)		
	DE (*n* = 120)	OMA (*n* = 46)	SUP (*n* = 70)
0	22 (18.3%)	31 (67.4%)	5 (7.1%)
0.01–0.49	39 (32.5%)	13 (28.3%)	51 (72.9%)
0.50–0.99	21 (17.5%)	2 (4.3%)	13 (18.6%)
1.00 or higher	38 (31.7%)	0 (0%)	1 (1.4%)

PGP9.5, Protein gene product 9.5; DE, deep endometriosis; OMA, ovarian endometriomas; SUP, superficial peritoneal endometriosis. Note the anatomic types are not mutually exclusive (i.e. a single patient can have more than one anatomic type), thus precluding a direct statistical comparison between the three columns.

**Figure 4:**
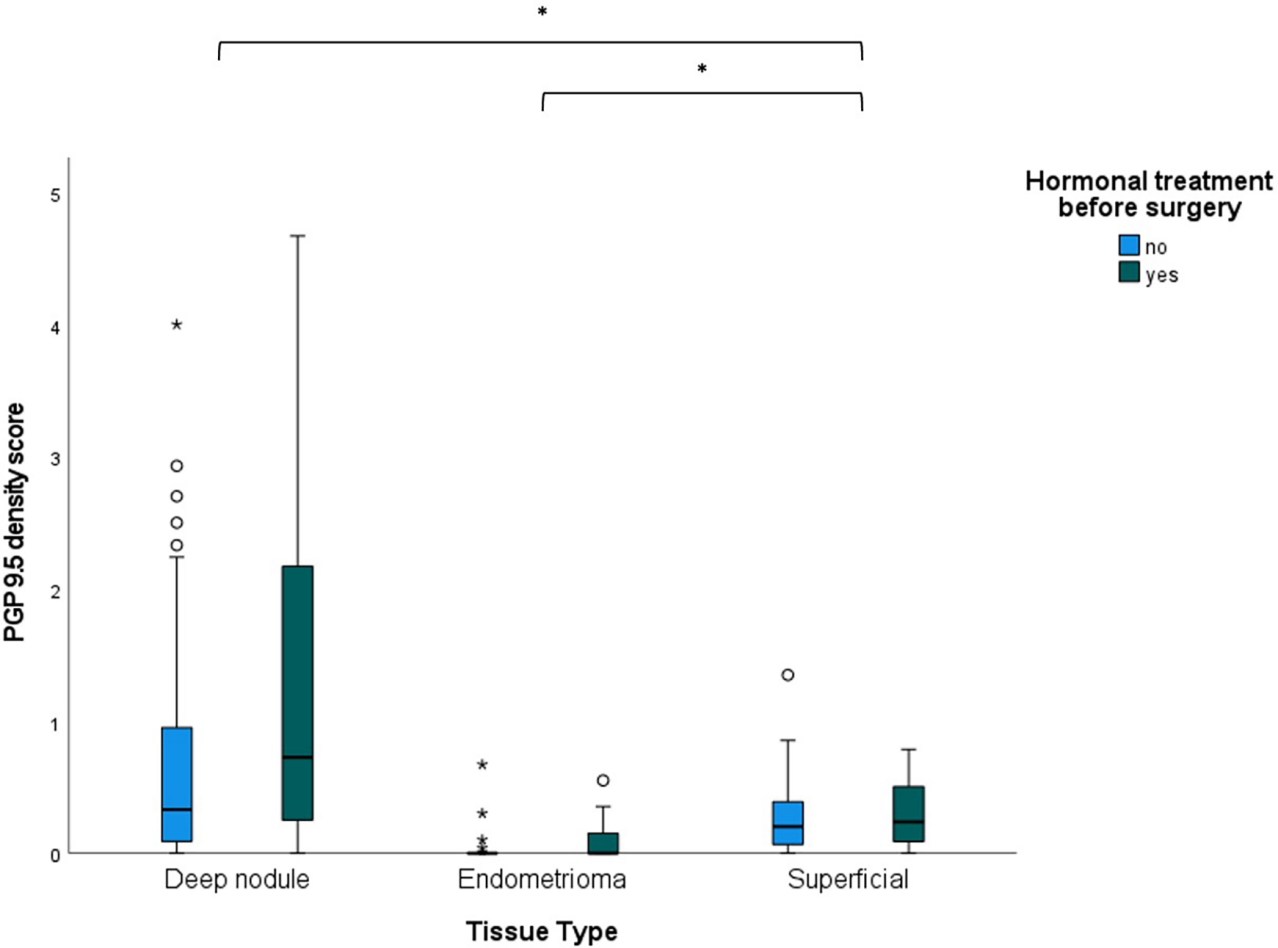
Boxplots of PGP9.5 nerve bundle density scores in different endometriosis tissue types (deep nodules, endomentriomas and superficial lesions), taking into account hormonal treatment before surgery. *p < 0.01.

Figure 4 graphically shows that PGP9.5 nerve density was higher in DE and SUP compared to OMA. GLMM controlling for hormonal treatment showed a significant difference in PGP9.5 nerve bundle density scores amongst the three anatomic types (DE, OMA, SUP) (X^2^ = 87.6, *P* < 0.001). In pair-wise comparisons with SUP as the reference group, PGP9.5 nerve bundle density scores were significantly higher in DE (estimate 1.111, 95% CI 0.793–1.430, *p* < 0.001) compared to SUP, and significantly lower in OMA compared to SUP (estimate −0.858, 95% CI −1.377 to −0.339, *p* = 0.001).

## Discussion

In this study, we utilized a standardized protocol to assess PGP9.5 nerve bundle density in 236 samples from 121 participants in an ongoing prospective cohort study. Using this protocol, PGP9.5 nerve bundle density in endometriosis lesions can be computed in a semi-automated fashion. The intra observer and inter observer reliability was excellent for PGP9.5 nerve bundle density. GLMM showed that PGP9.5 nerve bundle density varied between anatomic types (DE > SUP and OMA < SUP), taking into account the use of hormonal treatment before surgery.

Strengths of this study include the prospective design and the standardized and comprehensive clinical data collection. A limitation is that automated surface area measurement was not feasible in all samples. This was due to the density of the tissue in these samples, which appeared to be fatty tissue in a small minority of the samples (5.5%) with a very low signal density, thus not being picked up by the GA3 workflow. However, fatty tissue typically does not contain nerve bundles. We did not have the power to look at anatomic location within each anatomic type. Another significant limitation is that patient reported outcomes were not included in the current study.

Our analysis showed significantly higher PGP9.5 density score in DE compared to SUP. This is in accordance to the findings of a smaller retrospective study of 31 endometriosis patients undergoing surgery for endometriosis, which found significantly higher density in DE lesions than SUP lesions (9). In contrast, PGP9.5 nerves were less frequent in OMA compared to SUP, with nerve bundles absent in 67.4% of OMA's. In a retrospective study of 61 women with OMA who underwent surgery (cystectomy), PGP9.5 nerve fibers could be found in 31% of the participants but were absent in the remaining 69% (16). These results are comparable to the findings in our study.

While there is an increase in nerve bundle density around endometriosis compared to control tissue, the potential clinical implications of this “neuroproliferation” are still unclear (5). For example, some studies have shown an association between nerve density around endometriosis and deep dyspareunia, (7) which in turn correlates with NGF and interleukin-1β (IL-1β) expression by endometriosis epithelium/stroma (8). In contrast, another study compared pain groups of endometriosis based on dysmenorrhea and pelvic pain, and found no significant changes in NGF level in peritoneal fluid or when using this peritoneal fluid to induce neurite outgrowth from dorsal root ganglia (17). Given the multifactorial nature of endometriosis-associated pain, it is likely that substantial sample sizes will be needed to elucidate the specific role(s) of nerve density around endometriosis in different endometriosis pain symptoms, given the possibility of other pain generators that are potential confounders (18–21). The standardized protocol in this paper could be utilized in these larger studies to ensure that PGP9.5 nerve bundle density measurement is reproducible and reliable.

If future research shows that standardized measurement of nerve density around endometriosis has clinical relevance, it is possible that endometriosis could be phenotyped into neuroproliferative or non-neuroproliferative subtypes, a concept that has been incorporated into the classification of vulvar pain (vulvodynia) (5). Similarly, neuroproliferative subtyping could be one part of a future molecular classification of endometriosis.

## Conclusion

In summary, we present a carefully standardized protocol to quantify PGP9.5 nerve bundle density in endometriosis, with excellent intra observer and inter observer reliability. Further research is needed to externally validate in independent cohorts, and should focus on the relation between PGP9.5 nerve bundle density in different anatomic locations and with symptoms such as pain.

## Data Availability

The datasets presented in this article are not readily available because we do not currently have IRB/REB ethics approval for public sharing of raw data; the only exception would be via an an ethics approved collaboration with another researcher. Requests to access the datasets should be directed to paul.yong@vch.ca.
